# Frictional resistance calculation and jacking force prediction of rectangular pipe jacking

**DOI:** 10.1038/s41598-023-42189-9

**Published:** 2023-09-11

**Authors:** Chao Kong, Guoqing Guan, Song Gu, Zaiyan Zhou, Haiyan Wang

**Affiliations:** 1 School of Civil Engineering, Southwest University of Science and Technology, Mianyang, 610031 People’s Republic of China; 2 China Railway 15th Bureau Group City Construction Engineering Co. Ltd., Luoyang, 471000 People’s Republic of China; 3 College of Transportation Engineering, Nanjing Tech University, Nanjing, 211816 People’s Republic of China

**Keywords:** Civil engineering, Mechanical engineering

## Abstract

In practical engineering, whilst estimating the jacking force of rectangular pipe jacking using an empirical formula, the results obtained from said formula deviate from reality and manifest inadequate engineering guidance. The equations governing the applied force during the installation of rectangular pipe jacking have been derived for various contact states involving the interaction between the pipe, slurry, and soil. The distinct stress conditions in the pipe jacking process as well as the shear-friction mechanism between the pipe and the surrounding soil have been taken into account. The displacement control method is introduced to simulate the pipe–slurry–soil contact friction during the pipe jacking process in FLAC^3D^. Additionally, the pipe jacking behavior, pipe–slurry–soil contact frictional force, and variation law of the jacking force are also simulated. Mutual verification was carried out using the results obtained from field monitoring, numerical and theoretical. The findings are as follows: the established equations for calculating pipe jacking force are highly applicable across various conditions of pipe–slurry–soil contact, and the outcomes derived from theoretical formulas align remarkably well with those obtained through field monitoring and numerical simulation. During the jacking process, the sidewalls exhibit initial partial sliding followed by a complete movement as the jacking force intensifies and subsequently diminishes, eventually attaining stability during the behavior adjustment phase. Moreover, the bottom pipe–soil contact is the most common situation in actual construction.

## Introduction

Pipe jacking represents a frequently utilized trenchless technology extensively applied in underground passageways and municipal engineering projects. The jacks are required to provide jacking force in the pipe jacking process, the estimation of jacking force largely depends on engineering experience. The magnitude of the jacking force is directly proportional to both the precast strength of the pipe section and the design strength of the reaction wall. The accuracy of predicted jacking force is closely tied to the project's safety and cost-efficiency.

Frictional resistance around the pipe constitutes the primary factor influencing jacking force. Numerous studies have been undertaken to elucidate the correlation between jacking force and frictional resistance. By contemplating the potential impact of pipe–soil friction and pipe–slurry frictional resistance, the jacking force calculation model was divided into three distinct parts. The initial segment solely analyzed pipe–soil contact, asserting that frictional resistance equated to the earth pressure on the pipe jacking multiplied by the pipe–soil friction coefficient^1–5^. In the subsequent phase, exclusive attention was directed towards pipe–slurry contact, involving an assessment of thixotropic slurry as a power-law fluid and utilizing a fluid flat model to compute the pipe–slurry shear stress^6,7^. The final part encompassed both pipe–soil and pipe–slurry contact analyses, presuming that thixotropic slurry could not entirely envelop the pipe sections, thus maintaining contact with both soil and slurry during pipe jacking^8–12^. Nonetheless, these studies face two challenges: (1) Most investigations have centered on circular jacking pipes, with limited exploration of rectangular jacking pipes; (2) The consideration of thixotropic slurry in jacking force analyses has been oversimplified and diverges from practicality, rendering it unfeasible.

The utilization ratio of rectangular pipe jacking is 20% higher than that of circular pipe jacking for the same cross-sectional area, which makes rectangular pipe jacking more widely used in engineering. However, there is currently no suitable method to calculate the jacking force of rectangular pipe jacking tunnels. The design calculations for actual engineering projects mainly rely on circular tunnel pipe jacking methods, which lack accurate predictions for jacking force due to poor construction guidance.

In this study, considering the different contact states of pipe–slurry–soil in practical engineering, a jacking force calculation model and formula were proposed based on the analyses of pipe–slurry and pipe–soil contacts. The FLAC^3D^ finite element software and displacement control method were adopted to simulate pipe jacking by studying the jacking posture and jacking force of the pipe jacking, after which the simulation results were compared with the theoretical results. Then, using these cases, the formula derived from this study was compared with the existing formula. Finally, this formula was applied to other pipe jacking projects to verify its universality.

## Prediction formulas of jacking force

**Figure 1:**
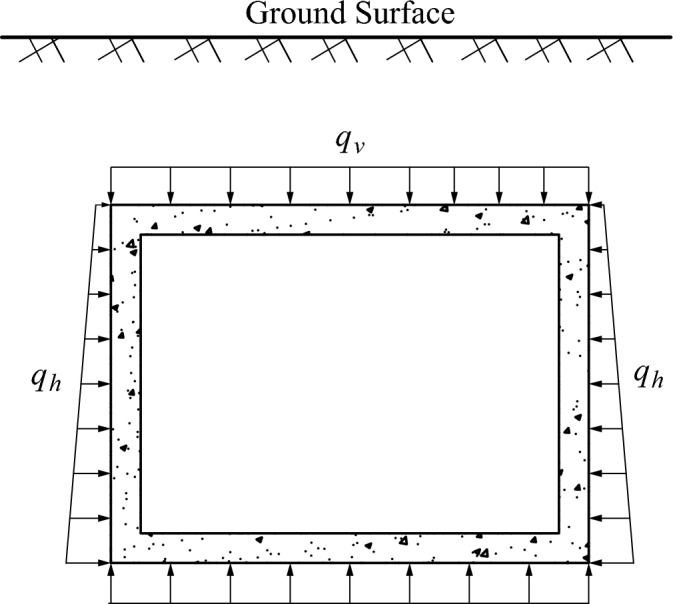
Schematic diagram of the cross section of the jacking pipe.

**Figure 2:**
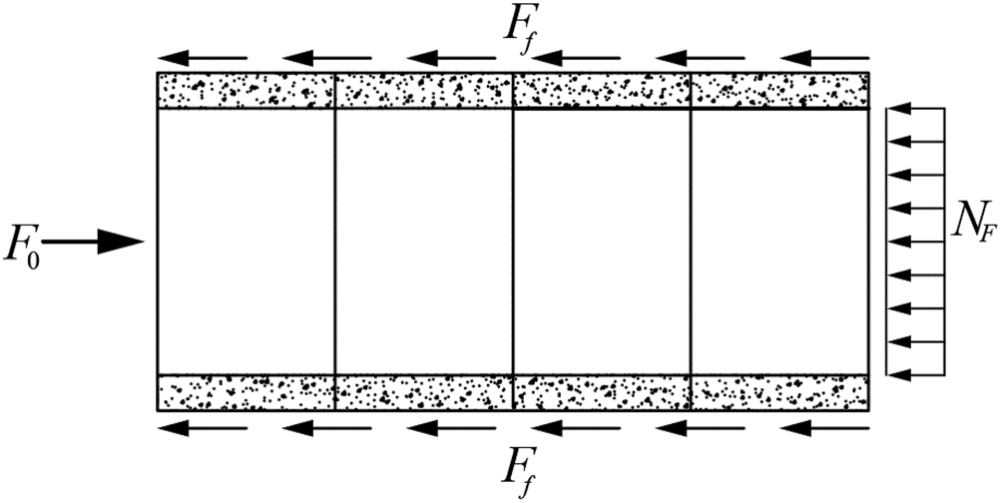
Stress diagram of pipe jacking.

Jacking pipe jacked in the soil was subjected to the vertical and horizontal pressure of the soil, as illustrated in Fig. 1.The jacking force is composed of the lateral frictional resistance of the pipe–slurry–soil and the frontal resistance, as illustrated in Fig. 2, and it can be calculated using Eq. (1). The lateral frictional resistance has a greater influence, and the frontal resistance is related to the stability of the tunnel face, which can be considered as a fixed value^13^.

1 $$F_{0} = F_{f} + N_{F}$$

where $F_{0}$, $F_{f}$, and $N_{F}$ represent the jacking force, lateral frictional resistance, and the frontal resistance.

The Chinese standard adopts the calculation formula of the circular pipe jacking force^3^ to estimate the jacking force of the rectangular pipe jacking, as expressed by the following equation:

2 $$F_{0} = k\left( {\pi DL{\text{N}}_{{\text{a}}} } \right) + N_{{\text{F}}}$$

where N_a_ represents the average frictional resistance per pipe joint, recommended to be within the range of 7–12 kN/m^2^. *N*_F_ represents the frontal resistance. D and L represent the diameter of the pipe segment and the jacking distance, respectively. k represents the safety factor, which is determined based on the actual situation and is generally in the range of 1.2 to 1.5.

Equation (2) is used to calculate the jacking force of the circular pipe jacking by the Japan Micro-Tunnelling Association^14^, and it is also applied to calculate the jacking force of the rectangular pipe jacking.

3 $$F_{0} = \pi DL\tau_{a} + N_{{\text{F}}}$$

where D and L represent the diameter of the pipe segment and the jacking distance, respectively. $\tau_{a}$ represents the lateral frictional resistance, which can be calculated using Eq. (4). *N*_F_ represents the frontal resistance, which can be calculated using Eq. (5).

4 $$\tau_{a} = {\text{c}} + f\sigma$$

where c*,* $\sigma$, and *f* represent the soil cohesion, earth pressure, and pipe–soil friction coefficient, respectively.

5 $$N_{{\text{F}}} = (P_{e} + P_{w} )(D/2)^{2} \pi$$

where *P*_*e*_ and *P*_*w*_ represent the unit area pressure of the excavation face and the grouting pressure, respectively.

Considering the influence of the thixotropic slurry^12^, the formulas for calculating the rectangular jacking force were proposed based on the power-law fluids and fluid-plate models, as expressed in Eq. (5).

6 $$F_{0} = 2\tau_{1} L(b + h) + 13.2 \times A \times N^{\prime }$$

where $\tau_{1}$ is the shear stresses of pipe–slurry contact, which can be calculated by $\tau_{1} = K(v/2\varepsilon )^{m}$; $v$ and $m$ are parameters of the mud, which can be measured by a viscometer. K is the viscosity constant, and $\varepsilon$ represents the mud cake thickness. A (m^2^) is the area of excavation head, *a* (m) and *b* (m) indicate the cross-sectional parameters of the pipe jacking. $N^{\prime }$ is an empirical factor, which equals to 1.0 for clayed soil, 2.5 for sandy soil and 3.0 for gravel soil^14^.

By conducting a comparative analysis of the calculation formulas, the following deductions can be made: (1) There is no formula for rectangular pipe jacking in the code of Asian countries, and the circular pipe jacking calculation formula is still used to calculate the rectangular jacking force, which is of poor applicability. (2) Both calculation formulas (1) and (2) ignore the influence of thixotropic mud on the lateral frictional resistance, which may lead to overestimated results. (3) Although calculation formula (3) takes into account the influence of thixotropic mud, it requires more calculation parameters and difficult to obtain accurate values, resulting in poor practical application effect.

## Calculation of rectangular pipe jacking force based on pipe–slurry–soil contact state

### Pipe–slurry–soil contact state classification

**Figure 3:**
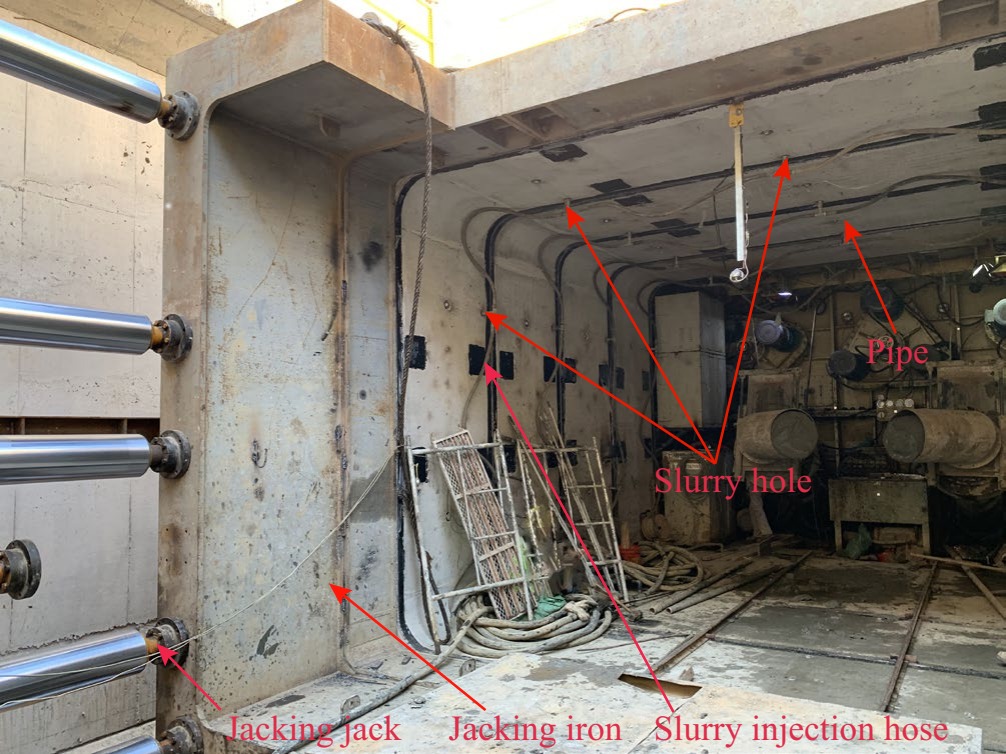
Layout of site grouting.

In the process of the tunneling, a layer of mud jacket is typically injected between the pipe and the surrounding soil to mitigate frictional resistance. The desired outcome is to achieve a uniform and complete filling of the mud jacket between the pipe and the soil, creating a continuous "mud jacket." And the pipe exclusively slide within the confines of the mud jacket. However, due to the influence of grouting methods, equipment, and other factors, the onsite grouting process, as shown in Fig. 3, often fails to achieve uniform coverage of the mud jacket around the pipe. Consequently, this leads to varying contact state between pipe jacking, mud jacket, and surrounding soil.

**Figure 4:**
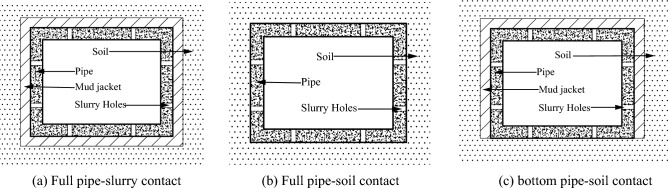
Different contact conditions.

In the ideal state, the mud jacket fully envelops the pipe during tunneling, and the pipe is in full pipe–slurry contact, as shown in Fig. 4a. In extreme situations such as surrounding soil collapse or wrong grouting process, the mud jacket fails to form, and the pipe is in full pipe–soil contact, as shown in Fig. 4b. In actual construction, due to factors such as gravitational compression and uneven grouting, the pipe often experience a "pipe–soil" contact state at the bottom, as shown in Fig. 4c. Therefore, it is essential to calculate and analyze the frictional resistance based on the different pipe–slurry–soil contact states during the construction process.

### Calculation of the lateral frictional resistance

In shallow tunnels, the depth of the tunnel is relatively small, and the stress distribution is primarily influenced by the overlying soil and surface loads. The stress state in shallow tunnels is typically dominated by vertical stresses due to the weight of the soil above the tunnel, resulting in a relatively simpler stress state. The arching effect is typically less significant due to the limited depth. The tunnel arch effect is more pronounced in deeper tunnels where the overlying soil or rock has a greater capacity to redistribute stresses^15–18^. The calculation is segregated into two cases based on the depth of burial: no arching effect and arching effect.

#### No arching effect

**Figure 5:**
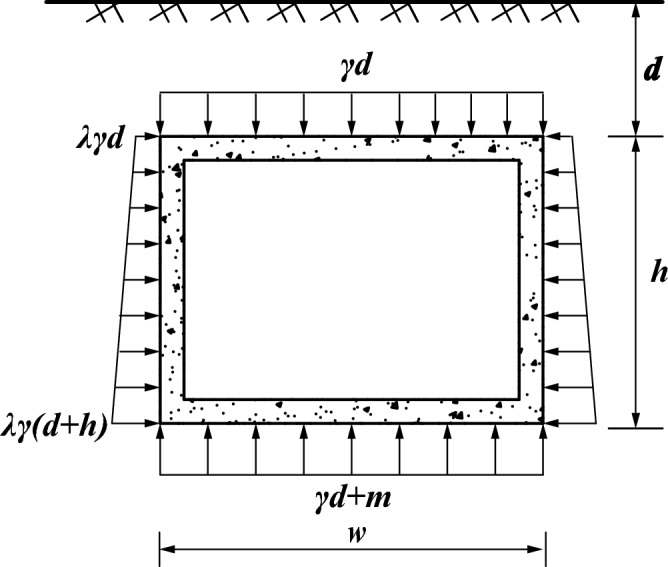
Earth pressure of pipe jacking.

The arching effect, which was in the layer of the cohesive and backfill soil, was not considered. The calculation diagram of the earth pressure is illustrated in Fig. 5. The earth pressure was calculated by the soil column theory^19^. Vertical earth pressure was calculated by $q_{v} = \gamma d$ while horizontal earth pressure was calculated by $q_{h} = \lambda \gamma \;(d + h)$, in which $\lambda$ represents the lateral pressure coefficient, $\gamma$ represents the density of soil. The base reaction force was calculated by $q_{r} = \gamma d + {\text{m}}$, in which *m* represents the weight of the pipe section per unit length.

##### Full pipe–slurry contact

The calculation formulas of jacking force are defined as follows:

7 $$F_{f} = fqA$$

where *F*_*f*_, *f*, q, A represent the lateral frictional resistance, friction coefficient, earth pressure on the pipe and contact area.

By plugging the values of the friction coefficient between the pipe and the slurry (*f*_*slurry*_), the earth pressure ($q_{v}$, $q_{{\text{h}}}$, $q_{{\text{r}}}$) and contact area (wl, 2hl, wl, l is the jacking distance) acting on the top, sides, and bottom of the pipe into Eq. (7):

8 $$F_{f} = f_{slurry} l\left[ {\left( {2\gamma d\left( {\lambda h + {\text{w}}} \right)} \right) + \lambda \gamma h^{2} + {\text{m}}} \right]$$

And the jacking force of full pipe–slurry contact:

9 $$F_{0} = f_{slurry} l\left[ {\left( {2\gamma d\left( {\lambda h + w} \right)} \right) + \lambda \gamma h^{2} + m} \right] + N_{{\text{F}}}$$

where d, h, w, *N*_*F*_ represent the depth of burial, height of pipe, width of pipe and the frontal resistance.

##### Full pipe–soil contact

The calculation formulas for this condition are expressed as follows:

By plugging the values of the friction coefficient between the pipe and the slurry ($f_{{{\text{soil}}}}$), the earth pressure ($q_{v}$, $q_{{\text{h}}}$, $q_{{\text{r}}}$) and contact area (wl, 2hl, wl, l is the jacking distance) acting on the top, sides, and bottom of the pipe into Eq. (7), And the jacking force in the full pipe–soil contact condition:

10 $$F_{0} = f_{soil} l\left( {2\gamma dw + \lambda \gamma h^{2} + 2\lambda \gamma dh + m} \right) + N_{{\text{F}}}$$

##### Bottom pipe–soil contact

When the mud jacket is not formed at the bottom, the contact is simplified to the pipe–soil contact, and the jacking force can be calculated as expressed below:

11 $$F_{0} = l\left[ {f_{slurry} \left( {\gamma db + \lambda \gamma h^{2} + 2\lambda \gamma dh} \right) + f_{soil} \left( {\gamma db + w} \right)} \right] + N_{F}$$

#### Arching effect

**Figure 6:**
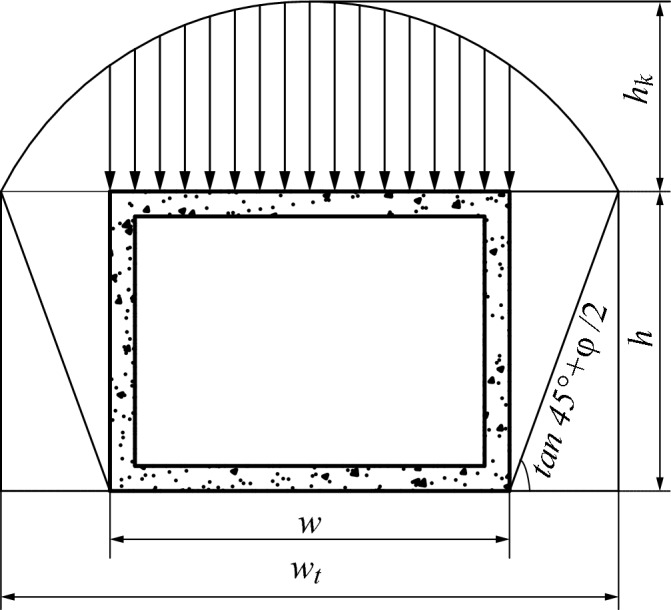
Schematic diagram of Promojiyfakonov arch theory.

The arching effect, which was in the layer of the cohesive and backfill soil, was considered. In this study, the Promojiyfakonov arch theory was used to calculate the soil pressure on the pipe jacking^20^. The soil above the arch is in a self-equilibrium state and has no force on the pipe jacking. Only the force of the soil below the arch was considered. The calculation diagram of the earth pressure is illustrated in Fig. 6.

According to Promojiyfakonov arch theory the collapse width was calculated by $w_{t} = w/2 + h\tan \;(45^\circ - \phi_{0} /2)$ while the height of the collapsed soil volume was calculated by $h_{k} = w_{t} /\xi$ in which $\xi$ represents the Protodyakonov's coefficient calculated by $\xi = \tan \phi + c/\sigma$. Which *c* represents the cohesion, $\sigma$ represents the normal stress at shear failure and $\phi$ represents the pseudo friction angle.

##### Full pipe–slurry contact

The jacking force can be calculated as expressed below:

12 $$F_{0} = f_{slurry} l\left[ {\frac{{2\gamma w_{t} }}{\xi }\left( {w + hK_{a} } \right) + mw + \gamma h^{2} K_{a} - 4hc\sqrt {K_{a} } } \right] + N_{F}$$

where *K*_*a*_ represents the active earth pressure coefficient.

##### Full pipe–soil contact

The jacking force can be calculated as expressed below:

13 $$F_{0} = f_{soil} l\left[ {\frac{{2\gamma w_{t} }}{\xi }\left( {w + hK_{a} } \right) + mw + \gamma h^{2} K_{a} - 4hc\sqrt {K_{a} } } \right] + N_{F}$$

##### Lower pipe–soil contact

The jacking force can be calculated as expressed below:

14 $$P = f_{sluury} l\left[ {\frac{{\gamma w_{t} }}{\xi }\left( {w + hK_{a} } \right) + \gamma h^{2} K_{a} - 4hc\sqrt {K_{a} } } \right] + f_{soil} lw\left( {\frac{{\gamma w_{t} }}{\xi } + m} \right) + N_{F}$$

### Calculation of frontal resistance

Generally, when the diameter of the pipe jacking, layer, pipe jacking machine type, and buried depth of the pipe are determined, the frontal resistance of the pipe jacking machine is a fixed value. The frontal resistance is mainly composed of the resistance on the cutter head ($F_{1}$) and the pressure on the cutting surface ($F_{2}$). The frontal resistance can be calculated by Eq. (15).

15 $$N_{F} = F_{1} + F_{2} = \eta \pi R_{1}^{2} P_{1} + AP_{2}$$

where $\eta$, $R_{1}$, $P_{1}$, A and $P_{2}$ represent the excavation coverage of the cutter head, radius of the cutter head, unit area resistance of the cutter head (taken at 0.15 MPa for the cohesive soil layer and 0.30 MPa for the gravel layer), excavation area and the earth pressure, respectively.

### Friction coefficient

When the pipe was in contact with the slurry, the friction coefficient of the pipe–slurry was represented by $f_{slurry}$. When the pipe was in contact with the soil, the pipe–soil friction coefficient was $f_{soil}$. The friction coefficients are determined by the respective friction angles, as shown in Eq. (16). The friction coefficient can be selected according to Table 1^13^. In this study, the friction coefficient of the pipe–soil friction coefficient ($f_{soil}$) is 0.25 while the pipe–slurry friction coefficient ($f_{slurry}$) is 0.15.

16 $$\begin{aligned} & f_{slurry} = \tan \;\delta_{slurry} \\ & f_{soil} = \tan \;\delta_{soil} \\ \end{aligned}$$

where $\delta_{slurry}$, and $\delta_{soil}$ represent friction angle of the pipe–slurry, and friction angel of the pipe–soil, respectively.

**Table 1:** Pipe–soil–slurry friction coefficient.

Type of contact	Friction coefficient
Sand–concrete	0.3–0.4
Clay–concrete	0.2–0.3
Fluid–concrete	0.1–0.3

## Numerical simulation

### Model and parameters

**Figure 7:**
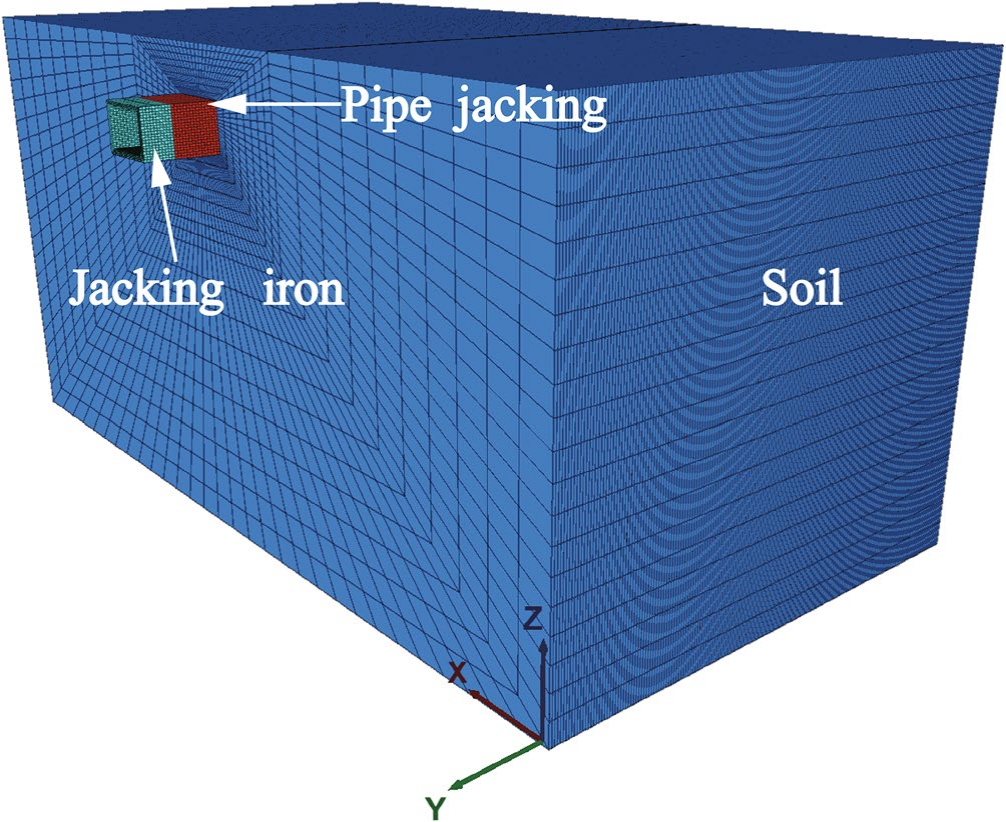
Numerical simulation model.

The finite difference software, FLAC^3D^^21^, has been used for numerical simulation of the exit of the Juhua Station of the Kunming Metro Line 4 case, as shown in Fig. 7. The X-axis and Z-axis are in the cross-section perpendicular to the tunnel alignment (Y-axis). The dimension of the model is 110 m × 505 m × 53 m (X × Y × Z). Each boundary is fixed in the direction perpendicular to it, except the top boundary where the pressure caused by the gravity is applied. The burial depth of the tunnel is 5 m. The pipe jacking structure adopted a precast reinforced concrete pipe section with the strength of C50, its overall dimension was 6.9 × 4.9 m, and the thickness and length of the pipe wall were 0.45 m and 1.5 m, respectively.

The soil in the model adopted the Mohr–Coulomb constitutive model, whereas the pipe jacking adopted the elastic constitutive model. The parameters of the soil and pipe jacking are presented in Table 2. Given the shallow burial depth of pipe jacking in the project area, there is no presence of groundwater. In addition, the influence of groundwater is extremely complex, so the influence of groundwater is not considered in order to simplify the calculation.

**Table 2:** The physical and mechanical parameters of the materials.

Properties	Soil mass Silt clay	Pipe Precast reinforced concrete(C50)
Unit weight (kN/m^3^)	20	25
Modulus of elasticity (E) (MPa)	13	34,500
Poisson’s ratio	0.35	0.20
Cohesion (kPa)	22	–
Friction (°)	15	-

### Contact surface

#### Working principle of the contact surface

In geotechnical engineering, two interfaces with large differences in stiffness are usually connected via interface elements. An interface element establishes a connection with the target surface through the contact surface nodes while the normal force on the contact surface is determined by the orientation of the target surface. During the calculation, the absolute normal penetration amount and relative shear velocity of the contact and target surfaces are obtained, whereas the normal and tangential forces are obtained by the constitutive equation of contact surface. When the contact surface is elastic, the normal and the tangential forces can be obtained by Eqs. (17) and (18).

17 $$F_{n}^{(t + \Delta t)} = k_{n} u_{n} A + \sigma_{n} A$$

18 $$F_{si}^{(t + \Delta t)} = F_{si}^{(t)} + k_{s} \Delta u_{si}^{(t + 0.5\Delta t)} A + \sigma_{si} A$$

where $F_{n}^{(t + \Delta t)}$ and $F_{si}^{(t + \Delta t)}$ indicate the normal force and the tangential forces of $t + \Delta t$, respectively; $u_{n}$ and $\Delta u_{si}$ represent the absolute displacement of the contact surface node penetration to the target surface and relative shear displacement increment, respectively;$\sigma_{n}$ and $\sigma_{si}$ indicate the additional normal and tangential stress caused by the initialization of contact surface, respectively; while $k_{n}$ and $k_{s}$ respectively indicate the normal and tangential stiffness of the contact surface.

**Figure 8:**
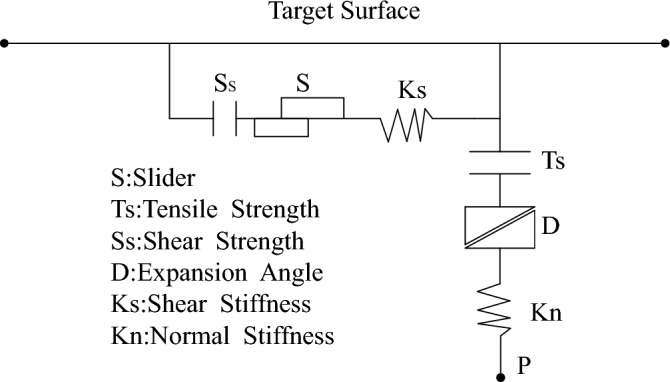
Schematic diagram of interface element.

Figure 8 presents the tangential stress required to slide the Coulomb constitutive contact surface, which can be calculated by the equation expressed below:

19 $$F_{s\max } = c_{if} A + \tan \phi_{if} \left( {F_{n} - uA} \right)$$

#### Contact surface parameters

To simulate the pipe–slurry–soil interaction, a contact surface was established between the pipe and soil, and the contact parameters were altered to simulate different pipe–soil contacts, including the full pipe–slurry contact, the full pipe–soil contact and bottom pipe–soil contact. Table 3^22^ presents the parameter characteristics of the contact surface.

**Table 3:** Contact surface parameters.

Types	kN (N/m^3^)	Ks (N/m^3^)	φ (°)	c (kPa)
Full pipe–soil contact	1e5	1e6	14	150
Full pipe–slurry contact	1e5	1e6	9	0

### Computational method

The force control method is frequently used in simulating the jacking process, enabling the analysis of interactions between the pipe, mud, and soil, which offers valuable guidance for construction^23^. However, this method requires the input of actual or theoretical jacking forces into the numerical model, followed by multiple iterations to adjust the forces until the pipe reaches the intended position. Consequently, it becomes time-consuming. In response to the limitations of the force control method, a refined simulation approach for pipe jacking has been developed, based on the principles of the displacement control method.

**Figure 9:**
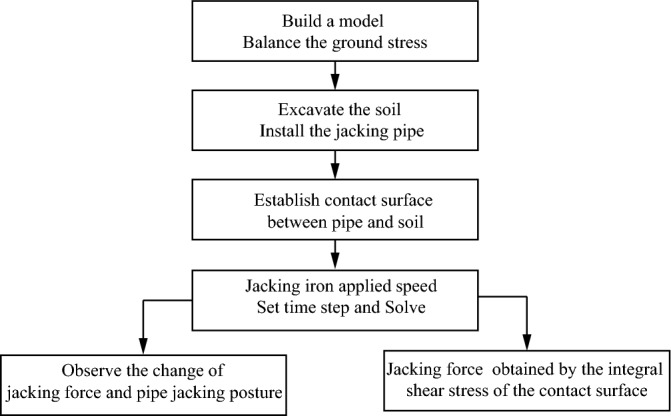
Simulation process of displacement control method.

**Figure 10:**
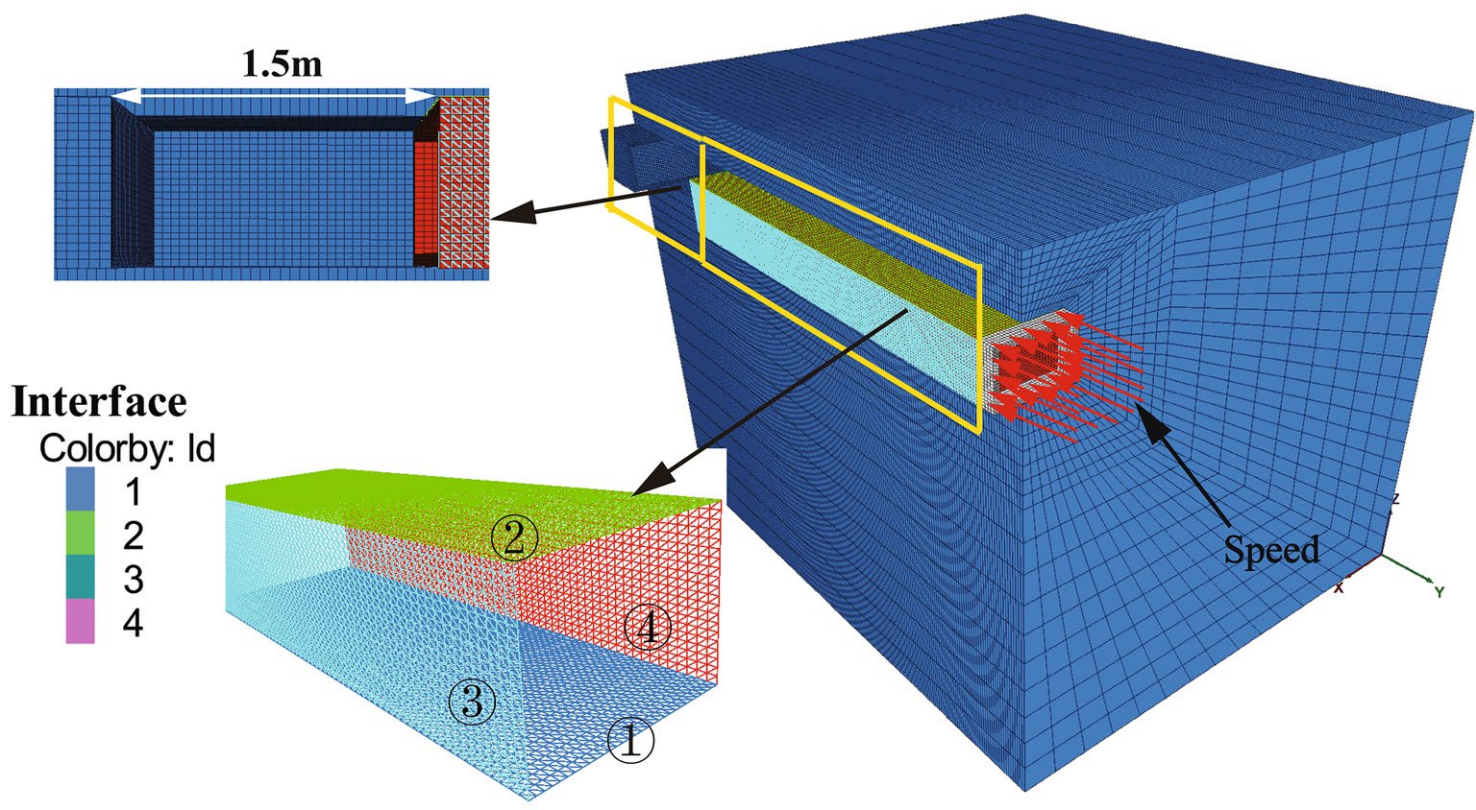
Schematic diagram of displacement control method.

In the displacement control method, the required time step is determined by considering the known parameters of jacking distance and speed. During the simulation, the pipe's velocity is assigned, and the time steps of the solution are adjusted until the pipe jacking reaches the predetermined location, as shown in Fig. 9. Subsequently, the jacking force can be calculated by integrating the obtained shear stress during the pipe jacking process. The simulation sequence of the displacement control method is illustrated in Fig. 10.

### Numerical results

#### Mechanical behavior of the contact surface during jacking

**Figure 11:**
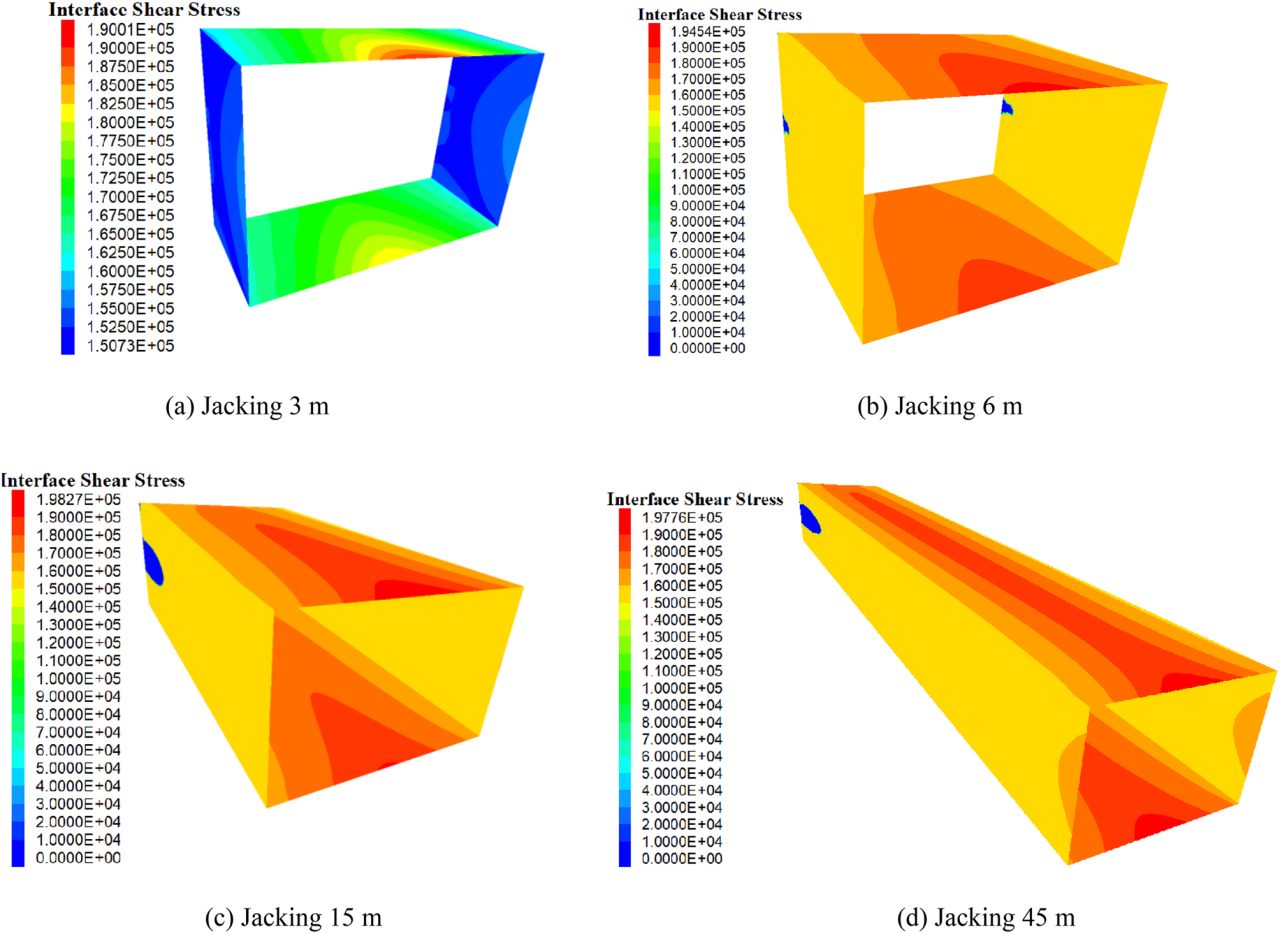
Shear stress distribution of contact surface.

**Figure 12:**
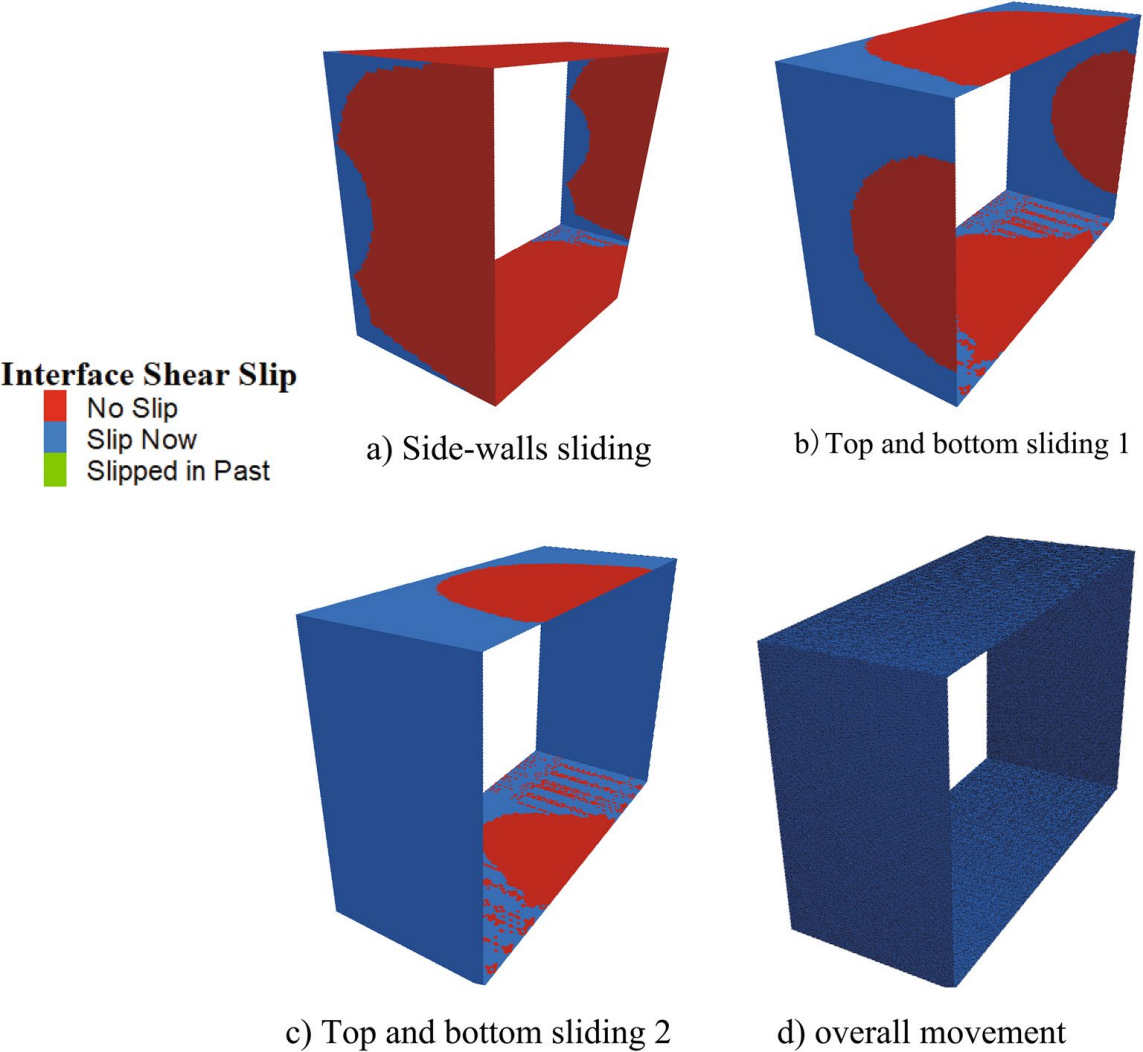
Shear sliding change of contact surface.

In order to determine the sliding tendency and mechanical behavior of the contact surface during jacking, the pipe–soil contact is taken as an example. The distribution of the shear stress of the contact surface is shown in Fig. 11, in which the maximum shear stress at the top and bottom parts of the pipe is higher than that of the side-wall. This occurs because the earth pressure on the top and bottom parts is higher than that of the side-wall, which results in a higher frictional resistance as the maximum shear stress increases with an increase in the jacking distance. During the calculation, at the same section of the pipe jacking, the contact surface of the side-walls first slide tangentially, followed by the top, and then the bottom, as illustrated in Fig. 12. This suggests that, under the jacking force, the side-walls first reach the maximum static friction and slide, followed by the top and then bottom, after which the pipe jacking is finally pushed forward as a whole.

The material properties of the jacking pipe, such as its stiffness, strength, and flexibility, directly affect the jacking force. Stiffer and stronger pipes can resist deformation and may require higher jacking forces. If the pipe experiences bending or deformation due to uneven soil conditions or misalignment, it can increase the jacking force. The maximum deformation of the jacking pipe in this calculation is only 10 mm, so its influence on the jacking force is small.

#### Pipe–slurry–soil contact

**Figure 13:**
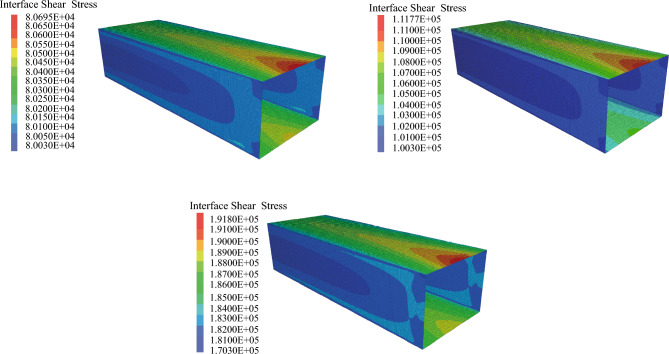
Shear stress distribution on different contact surfaces.

Figure 13 illustrates the distribution of shear stress among various contact surfaces (pipe–slurry–soil) when the jacking distance is 20 m. It is evident that the maximum shear stress rises with the increase in the pipe–soil contact area. The continuous increment in maximum shear stress from full pipe–slurry contact to full pipe–soil contact suggests that the utilization of thixotropic slurry from Table 4 can effectively reduce the jacking force. The calculation results show that the mud jacket can effectively reduce about 57% of the shear stress.

**Table 4:** Maximum shear stress on different contact surfaces.

Type of contact	Maximum shear stress (kPa)
Full pipe–slurry contact	80.69
Bottom pipe–soil contact	111.77
Full pipe–soil contact	191.80

#### Jacking force

**Figure 14:**
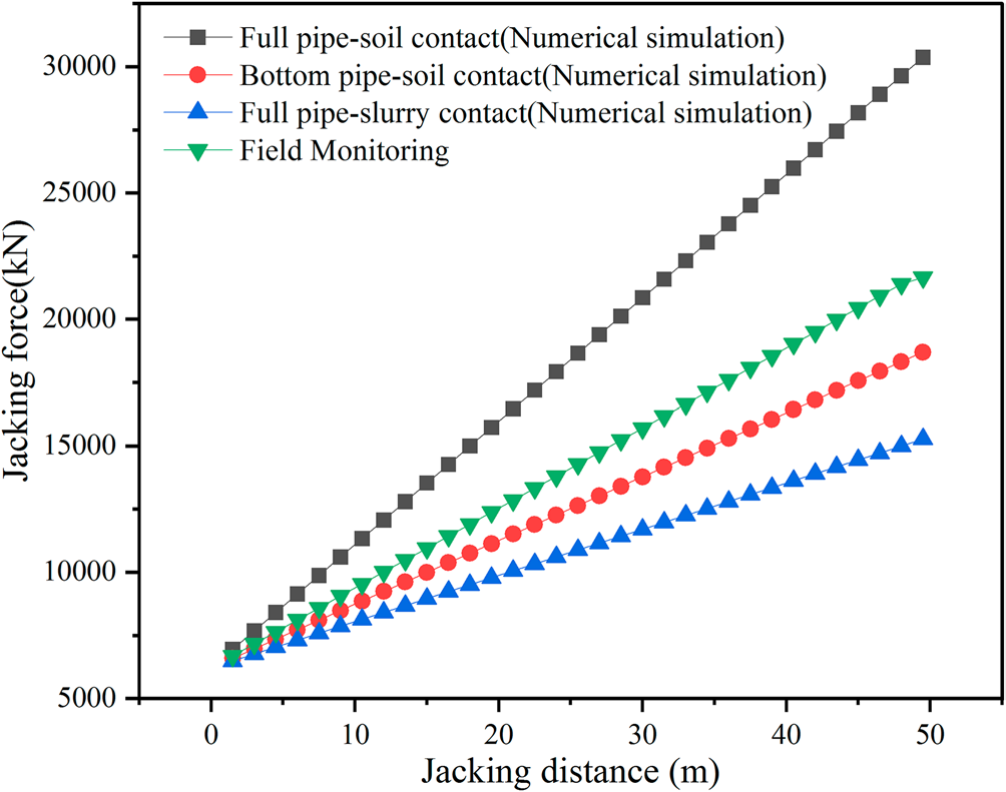
Comparison of numerical analysis and field monitoring.

The numerical simulation results are compared with the field monitoring data for the jacking force, as shown in Fig. 14. Monitoring data from Huahua Station of Kunming Metro, see section “Case 1: The Kunming Metro” for details. The jacking force increases linearly with the advancement of the jacking distance. The maximum jacking force occurs in the full pipe–soil contact condition, while the minimum jacking force is observed in the full pipe–slurry contact condition. The field jacking force lies between these two extremes. This pattern aligns well with the earlier analysis. The results indicate that the displacement control method effectively simulates the jacking process and accurately predicts the jacking force.

## Case study

### Case 1: the Kunming Metro

**Figure 15:**
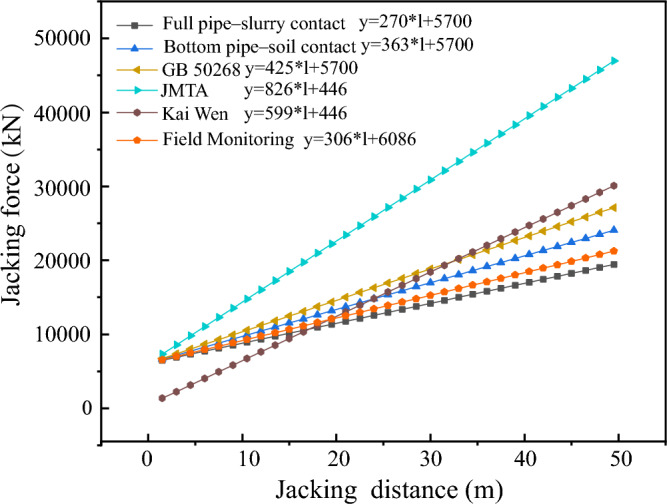
Jacking distance and jacking force.

The passageway of the Juhua Station on Kunming Metro Line 4 were constructed using pipe jacking techniques. The jacking distance extended approximately 50 m, with a burial depth of 5.0 m. The soil layers traversed by the pipe jacking project predominantly consist of silty clay. The dimensions of the pipe and the computational parameters are comprehensively detailed in Table 5. The jacking forces were computed based on the formulas presented in this study, as depicted in Fig. 15.

**Table 5:** Pipe jacking engineering parameters of Juhua Station.

Pipe jacking parameters	
Buried depth, d (m)	5
Section size (m)	Width = 6.9, Height = 4.9
Pipe section parameters	
Weight per unit length, w (kN/m)	247
Elastic modulus, E (GPa)	34.5
Poisson's ratio, μ	0.2
Thickness, t (m)	0.45
Jacking velocity, $v$ (cm/min)	2
Excavation pressure, $P_{s}$ (MPa)	0.2
Soil parameters	
Unit weight (kN/m^3^)	20
Internal friction angle, φ (°)	22
Cohesion, c (kPa)	15
Pipe–slurry friction coefficient, $f_{slurry}$	0.15
Pipe–soil friction coefficient, $f_{soil}$	0.25
Thixotropic slurry parameters	
Slurry viscosity, m ($Pa \cdot s$)	0.5
Slurry thickness, $\varepsilon$ (m)	0.06
Viscosity constant, K ($Pa \cdot s^{1/2}$)	1.31

Figure 15 illustrates the linear increase in jacking force with the advancement of the jacking distance. The jacking force calculated using the formulas proposed by Japan Mini Tunnel Association shows significant deviation from the jacking force observed through field monitoring. Similarly, the formulas proposed by Chinese National Standard also proves excessively large in comparison to the field monitoring data. The formulas introduced by Kai Wen show discrepancies with practical observations, leading to considerable variations in the jacking force that do not align with field monitoring results, it is impractically ideal to consider thixotropic slurry as power-law fluid as it fails to consider the effect of thixotropic slurry. Conversely, the jacking force prediction formulas proposed in this study exhibit the smallest deviation from the field monitoring jacking force, thereby offering superior guidance for on-site construction activities.

### Case 2: Shanghai Metro

**Figure 16:**
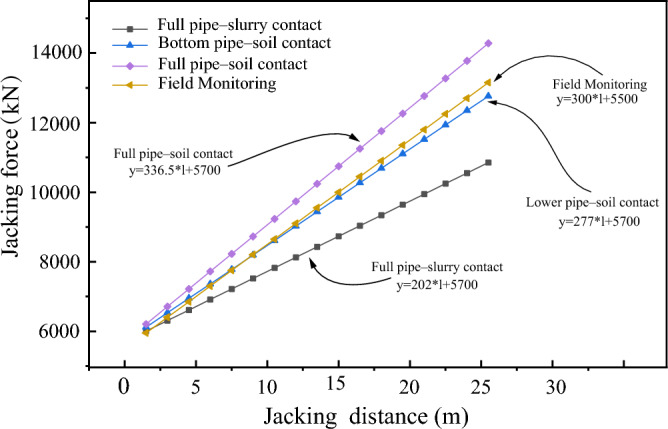
Theoretical jacking force and actual jacking force.

The passageway of the subway Station on Shanghai Metro Line 18 were constructed using pipe jacking techniques. The jacking distance extended approximately 25 m, with a burial depth of 3.5 m. The soil layers traversed by the pipe jacking project predominantly consist of silty clay. The dimensions of the pipe and the computational parameters are comprehensively detailed in Table 6. The jacking forces were computed based on the formulas presented in this study, as depicted in Fig. 16. The figure illustrates a close correspondence between the measured curve and the calculated curve. It suggests that the theoretical formula provides a more accurate estimation of the actual jacking force. Furthermore, the theoretical formula displays commendable generality, rendering it adaptable to diverse geological strata for predicting pipe jacking forces.

**Table 6:** Pipe jacking engineering parameters of Shanghai Metro.

Pipe jacking parameters	
Buried depth, d (m)	3.5
Section size (m)	Width = 6.9, Height = 4.2
Pipe section parameters	
Weight per unit length, w (kN/m)	266
Elastic modulus, E (GPa)	34.5
Poisson's ratio, μ	0.2
Thickness, t (m)	0.45
Jacking velocity, $v$ (cm/min)	2
Excavation pressure, $P_{s}$ (MPa)	0.15
Soil parameters	
Unit weight (kN/m^3^)	21
Internal friction angle, φ (°)	24
Cohesion, c (kPa)	12
Pipe–slurry friction coefficient, $f_{slurry}$	0.15
Pipe–soil friction coefficient, $f_{soil}$	0.25

## Conclusion

The priority of the pipe jacking project is the estimation of the jacking force. This estimation is interconnected with factors such as the design strength of the reaction wall, the strength of prefabricated pipe, the selection of jacks, and the jacking force during construction. To achieve precise jacking force estimation, the influence of thixotropic slurry was taken into account, leading to the proposal of a prediction model. This model is founded on the principles of earth pressure in pipe jacking, as well as the friction at the interfaces of pipe–soil and pipe–slurry contacts. Consequently, the following conclusions can be drawn:

1. Based on an analysis of various contact conditions, a formula for calculating the rectangular jacking force was proposed. In comparison with existing formulas, the formulation introduced in this paper is more realistic and comprehensively accounts for actual jacking force fluctuations.
2. Taking into account the pipe–slurry–soil contact, the displacement control method was used to accurately simulate the jacking process. The simulation outcomes were found to align with the actual scenario.
3. The results gleaned from numerical simulations depicted the pipe jacking process with initial partial and complete sliding of the side-walls. Meanwhile, the jacking force exhibited an increase surpassing the maximum static frictional force, followed by a subsequent decrease before eventually stabilizing.
4. By comparing the jacking force monitored on site, which was calculated by theory and simulated by the numerical method, the bottom pipe–soil contact is the most common situation in actual construction.

## Data Availability

All data generated or analysed during this study are included in this published article.
